# Involvement of gut microbiome in human health and disease: brief overview, knowledge gaps and research opportunities

**DOI:** 10.1186/s13099-018-0230-4

**Published:** 2018-01-25

**Authors:** Dachao Liang, Ross Ka-Kit Leung, Wenda Guan, William W. Au

**Affiliations:** 1 Division of Genomics and Bioinformatics, CUHK-BGI Innovation Institute of Trans-omics Hong Kong, Hong Kong SAR, China; 2 State Key Laboratory of Respiratory Disease, National Clinical Research Center for Respiratory Disease, First Affiliated Hospital of Guangzhou Medical University, Guangzhou, Guangdong China; 3 University of Medicine and Pharmacy, Tirgu Mures, Romania; 4 Shantou University Medical College, Shantou, China

**Keywords:** Microbiome, High throughput sequencing, Metagenomics, Human disease causation

## Abstract

The commensal, symbiotic, and pathogenic microbial community which resides inside our body and on our skin (the human microbiome) can perturb host energy metabolism and immunity, and thus significantly influence development of a variety of human diseases. Therefore, the field has attracted unprecedented attention in the last decade. Although a large amount of data has been generated, there are still many unanswered questions and no universal agreements on how microbiome affects human health have been agreed upon. Consequently, this review was written to provide an updated overview of the rapidly expanding field, with a focus on revealing knowledge gaps and research opportunities. Specifically, the review covered animal physiology, optimal microbiome standard, health intervention by manipulating microbiome, knowledge base building by text mining, microbiota community structure and its implications in human diseases and health monitoring by analyzing microbiome in the blood. The review should enhance interest in conducting novel microbiota investigations that will further improve health and therapy.

## What is microbiome?

Microorganisms often live in the form of a community. Furthermore, they can live in close association with complex organisms, such as plants and humans, by establishing commensal, ammensal, mutualistic, parasitic and/or pathogenic relationships with their hosts. The collection of such microorganisms is called microbiome or microbiota. Microflora has also been used but flora represents the kingdom Plantae therefore it is a misnomer.

In the original version, microbiome referred to the collection of microbes and their genomic contents. Microbiota indicated the microbial community in their host. But “microbiome” has frequently been used interchangeably with microbiota [1]. In this review, we focused mainly on bacterial microbiome with reference to either the collection of bacteria or their genomes, unless otherwise specified.

Microbiome can be found throughout the human body, ranging from the skin to the gut, and to previously considered as sterile environments such as the blood in circulation [2]. Various reports indicated that over 10,000 microbial species have been shown to occupy various parts of the human body [3, 4]. While diversity of microbes in the skin and vaginal sites are relatively low, great diversity can be found in other sites, e.g. the gut [6]. Consequently, impact of microbiome in human diseases and vice versa can be extensive. For example, chronic lung diseases can alter composition of lung microbiome which can subsequently influence host defense and immunity, thus leading to further exacerbation of the diseases [5]. Infection status has also been found to influence microbiome in the blood or the lung [6–9].

## What is the gut microbiome?

The gut microbiome is the genetic material of all the microbes, e.g. bacteria, fungi, protozoa and viruses which live on and inside the digestive tracts of humans and other animals, including insects. In this review, we focused on the human gut microbiome and on bacterial composition.

The human gut microbiome has co-evolved with its host for millennia and, therefore, has been extensively involved with a variety of essential activities in the host, e.g. digestion and nutrition [10, 11], detoxification and body defense [12], maturation of the host immune system [11] and disease mediation [13–17]. Consequently, a large number of microbes with high diversity can be found in the mammalian gut, with most of them being *Firmicutes* and *Bacteroidetes* [18]. Such observation has been confirmed in different populations: Europeans and Americans [19], Koreans [20], Africans [21, 22], Danish but not Chinese [23]. The diversity can have specific implications for disease in different populations. For example, European and Chinese citizens with type 2 diabetes had different gut microbiome compositions [24], with the Chinese having more diverse species [24]. However, the reason of the major difference between the two populations, e.g. as related to age, environmental and genetic factors needs further investigation [25].

With diverse microorganisms, the gut microbiome contains millions of different genes [19]. Some of them may be acquired from environmental bacteria [10], indicating their metabolic diversity and versatility. Accordingly, three major genera have been reported as enterotypes: *Bacteroides*, *Prevotella* and *Ruminococcus* in the human gut as observed from 22 Europeans, 13 Japanese and 4 Americans [26]. Interestingly, similar bacterial ecosystems were also identified in mice and chimpanzees [27–29]. Their content in the human gut has been reported to be mainly influenced by their evolving change in the host and much less by age, gender, body weight, or race [26, 30]. However, a recent study reported that diet had more influence on metabolome than microbiome. In another context, some studies reported that *Ruminococcus* was a major ecotype [30–32], including one which analyzed data from native populations from different countries [33]. In particular, *Enterobacteriaceae* belonged to the third major ecotype among Taiwanese [34]. However, these discrepancies need to be clarified with more attention to sample size, and sampling methods and variations.

There are two major categories of microbes in the gut microbiota: (1) autochthonous microbes that seem to reside on the epithelium of colonic mucosa, and (2) allochthonous microbes that transiently pass the lumen as part of the digesta [35]. The functional roles of these “residents” and “passengers” are believed to be very different. Indeed, the ratio of autochthonous to non-autochthonous microbes has been proven useful to assess cirrhosis progression [36].

In general, host diet and phylogeny contribute to modifying the composition of gut microbial community in mammals and other species [18, 37, 38]. Indeed, genome-scale metabolic modeling show that variations in the diet of the host significantly modified the composition of the three representative human gut bacteria (*B. thetaiotaomicron*, *E. rectale* and *M. smithii*) [39]. For example, alcohol is a common dietary modulator of intestinal microbiota, as shown in experimental animals and humans [40–44]. In return, different composition of the three representative human gut bacteria influenced host metabolism and related diseases.

There are many reports which indicate that host genetics played an important role in determining the composition of microbiome [15, 45–57]. For example, several susceptibility loci were shared by inflammatory bowel disease [16, 17, 52], with infectious mycobacterial and staphylococcal organisms. These associations were validated from studies using the Gene Co-expression Network Analysis [58]. Therefore, investigations on the relationships among susceptibility, microbiome composition and disease development can provide valuable evidence to develop disease prevention protocols.

In the gut, a typical microbial product is lipopolysaccharide (LPS) which are produced by Gram-negative bacteria [6, 59–62] and are transported with chylomicrons [63]. LPS has been shown to be strong stimulators of innate immunity in organisms from lower- to higher-order animals [64]. For peritoneal dialysis patients, LPS level is used as an important indicator for survival. Indeed, a retrospective study of 300 patients show that plasma bacterial DNA levels were positively correlated with serum C-reactive proteins and LPS levels, and negatively correlated with survival rates [7]. These results indicate that both plasma LPS and bacterial DNA levels can be used as indicators for systemic inflammation and for prognosis. Another important microbial product is Trimethylamine (TMA). The oxidation product of TMA by hepatic flavine monoxygenases, trimethylamine *N*-oxide (TMAO), has influence on morbidity of patients [65]. These observations indicate that localized microbiome can cause far-reaching consequences.

## Knowledge gaps and opportunities

**Fig. 1:**
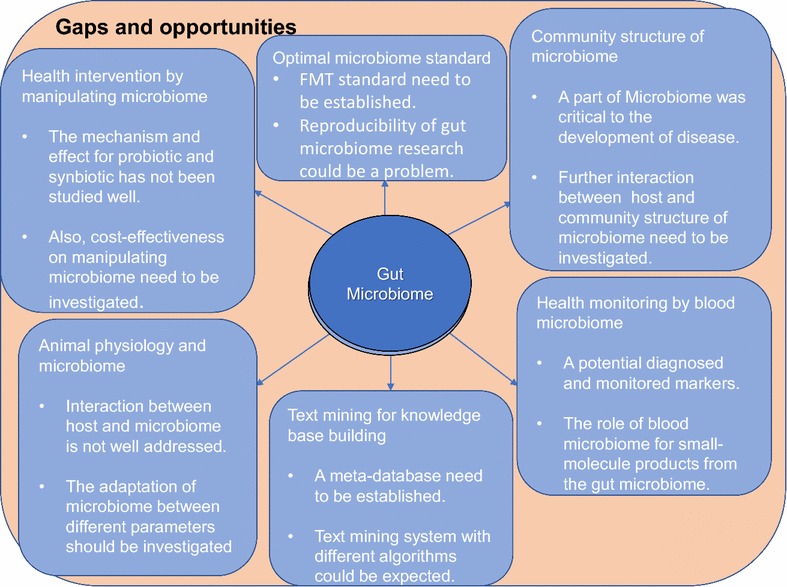
Summary of knowledge gaps and opportunities in current study

As mentioned earlier, stimulating observations in the new field of microbiome research has raised world-wide interest in the topic as well as many unanswered questions. Based on our review of the literature, we have identified several important issues and questions that may be useful for enhancement of novel research activities (Fig. 1).

### Microbiota and animal physiology

There have been suggestions to treat the gut microbiome as our second genome, even as one of our tissues or organs. If the latter would be the case, the parenchyma (main tissue) and stroma (“sporadic” tissues) would have to be defined. Furthermore, it is necessary to find out how the microbiota are connected with each other. Should they be grouped with our biological system such as the immune, digestive, nervous or endocrine system? Can we gain insights into the adaptation of microbiota to their host by comparing different microbiota in the tree of life to better understand their inter-relationships?

Reports have shown that several physiological functions were protected directly by specific microbes via their control of epithelial cell proliferation and differentiation, and via their production of essential mucosal nutrients [66]. In addition, microbiota can protect physiological functions indirectly. For example, a certain gut bacteria caused behavioral abnormality by host metabolome [67]. Furthermore, the main fermentation products of gut microbiome which are short-chain fatty acids (SCFA) acetate, propionate and butyrate plus gases provided external resources for metabolic activities [68]. In addition to microbiota itself, host parameters such as lifestyle, diet, drug usage, genetics and immune activities could influence the composition as well as different consequences of host physiology [69]. Consequently, there should be additional risk factors which can influence association between host physiology and microbiota.

### Optimal microbiome standard

Recently, a question was raised on reproducibility of investigations on gut microbiome research in experimental animals [70]. Some of the discrepancies can be due to biases on genetic and environmental factors [71]. For example, the lack of standardization in fecal microbiota transplantation protocol for multiple recurrent *Clostridium difficile* infection was a cause for reduced efficacy [72]. These concerns emphasize that environmental factors, rodent husbandry and treatment protocols must be standardized and be reproducible.

There have been recommendations to define microbiome composition inside an individual as a bacterial ecosystems [26], or “biomarkers” [33]. However, there are extensive variations from one individual to another. One example is the ratio of *Firmicutes* to *Bacteroidetes* (F:B) which was also affected by age [21, 73]. High F:B ratio has been reported to be associated with various pathological states [4, 11, 24, 74–76]. Therefore, it will be intriguing to investigate what would be the consequences if the F:B ratio is altered by adopting a vegetarian diet [77]. It should also be noted that liver and inflammatory bowel diseases can be associated with reduction in Firimutes but also with increase in *Bacteroidetes* [40, 78, 79]. In this context, what is the “normal” range of F:B ratio in a population and with respect to age? Consequently, what would be the standard composition of pathobiome [80] for healthy individuals?

### Health intervention by manipulating microbiota

In recent years, the identification of prebiotics (a non-digestible food ingredient that promotes growth of beneficial microorganisms in the intestine), probiotics (a microorganism introduced into the body for its beneficial qualities) and synbiotics (a mixture of prebiotic and probiotic which selectively promotes growth) has aroused strong research and commercial interests. However, no studies have been conducted to address clinical beneficiary from probiotics intervention [81]. Consequently, a main focus for probiotics research is to validate the benefit and the mechanisms for physiological effects via clinical trials.

With regards to probiotics, *Lactobacillus* and *Bifidobacterium* are most commonly used for investigations [82]. *Lactobacillus* has been considered an option for preventing antibiotic-associated diarrhea in children [83]. For example, *Lactobacillus casei* were reported to inhibit growth of *Helicobacter pylori* [84]. In addition, co-colonization of *Lactobacillus rhamnosus* GG and *Bifidobacterium lactis* Bb12 promoted innate immune responses to human rotavirus [84]. Other *Lactobacillus* strains were used as potential treatment options for non-alcoholic fatty liver disease [84], type 2 diabetes [84], and urinary tract [84] and HIV infections [85, 86]. Although *Lactobacillus* species have been used in dairy food production safely for a long time [87], species resolution sometimes matter because certain *Lactobacillus* strains are tolerant in a low pH circumstance [82, 88], and others are associated with diseases [24, 74, 75, 89–95]. Therefore, it is crucial to investigate what specific genes or factors can make the difference among these members in the *Lactobacillus* bacteria.

There are at least 9 *Bifidobacterium* species that are commonly identified in the human gut [63]. In pathological conditions such as colorectal cancer, inflammatory bowel disease, irritable bowel syndrome and obesity, the relative abundance of *Bifidobacterium* species either changed significantly or, as a whole, decreased substantially when compared with other gut microbiota [96–99]. However, *Bifidobacterium* species are also widely recognized for their beneficial effects. Like Lactobacilus, Bifidobacteria is also a popular probiotic. For example, different Bifidobacteria have been used as therapy to relieve symptoms in some respiratory diseases, such as asthma in infants with atopic dermatitis [100] and cedar pollinosis [101, 102]. Bifidobacteria can also interact with intestinal cells by regulating immunity and inflammatory gene expression. *B. longum* was reported to regulate TNF-α and IL-1α expression in ulcerative colitis patients [103]. Oral administration of *Bifidobacterium* also improved tumor-specific immunity [104, 105]. Therefore, further investigations on *Bifidobacterium*, *Lactobacillus* and other microbes as probiotics and standardization of their usage can bring major benefits to individuals and to the healthcare system. Further inquiries may include: are these two species the only members in the “probiome”, and which microbiota would bring beneficial health effects to the host?

The application of probiotics can be beyond conventional arena for harm reduction. For example, microbial transplantation was used to restore healthy gut microbiome and to improve therapeutic efficacy for recurrent *Clostridium difficile* colitis [106]. In another study, a single commensal microbe, segmented filamentous bacterium protected mice from pathogenic effects of *Citrobacter rodentium* [107]. Alcohol consumption is another example. While laws and regulations are commonly used to limit alcohol consumption, it is more difficult to control consumption than to provide supplement. In this case, alcoholic liver disease intervention was successful from the administration of prebiotics, probiotics or synbiotics that modulated the composition of healthy and pathogenic intestinal microbiota [41, 108, 109]. Therefore, more investigations can be focused onto interventions using prebiotics, probiotics, synbiotics or microbiome transplantation, and onto their cost-effectiveness.

### Knowledge base building by text mining

From our literature review, we recognized that several databases (e.g. Human Microbiome Project [HMP], The Integrative Human Microbiome Project [iHMP,], MetaHIT, Canadian Human Microbiome Initiative, and Australian Jumpstart Human Microbiome etc.) have been established. However, these databases are insufficient to archive the vast amount of published data. For example, there were no centralized resources which catalogued factors which influenced the gut microbiome composition. Having such a resource can be useful for conducting comprehensive and systematic data mining for host genetic, diet, disease, alcohol use, or other factors which can stimulate development of novel gut microbiome research. Moreover, statistical analyses, such as meta- or enrichment analyses can be made possible. To do this systematically, text mining is an option. Title and abstract data can be acquired in batches by software from the PubMed open access database. Sentence tokenization, and entity recognition for genes, microbiome, diet and environmental factors can also be conducted. Relationships among these factors (e.g. positive or negative effects) can be constructed and standard measures such as precision and recall can be used for evaluating text mining algorithms, with the accuracy as defined by two independent assessors. Recently, a free information system named @Minter can be used for analysis of abstracts and for inference microbial interactions base on Support Vector Machines with text-mining algorithm [110]. We expect that more and more platform or software can be used to concatenate different databases and to perform analysis with text-mining algorithm for increased efficiency of gut microbiome studies.

### Microbiota community structure and its implications in human diseases

Microbes have been shown to interact extensively with each other within the human bodies [50, 111, 112]. For example, the *Human Microbiome Project* (HMP) cohort study reported competition between *Porphyromonaceae* and *Streptococcus* species in dental plaques, and between *Prevotellaceae* and *Bacteroides* in the guts; but possible complementation between *Treponema* and *Prevotella* in dental plaques [112]. In addition, co-existence of *Candida* fungi and *H. pylori* in the gastric mucosa was critical to the development of non-ulcer dyspepsia, gastric ulcer and duodenal ulcer [113].

Interactions can also be beneficial to the host. Through the production of polysaccharide A (PSA), *Bacteroides fragilis* protected its host from the induction of colitis via *Helicobacter hepaticus* infection [114]. Indeed, PSA has recently been shown to activate intestinal sensory neurons and thereby modulated peristalsis [115]. Administration of *E. coli* O21:H^+^ also protected mice from muscle wasting which was induced by infections [52].

Although it has been well-acknowledged that interactions between host and microbiome can significantly influence health and modulate clinical outcomes, more detailed mechanistic investigations are needed to better understand the important interactions and the opportunity for interventions. Investigations into interactions can borrow concepts from a seminal study in human disease network [116]. The then-hypothesis was if each human disorder had a distinct profile of microbiome, or ‘the pathobiome’ [80], the human disease network would be subdivided into many single nodes which corresponded to specific disorders or to small clusters of a few closely related disorders. Likewise, if microbial genes which were linked by disorder association with encoded proteins that interacted in functionally distinguishable modules, then the proteins within such disease modules would more likely interact with one another than with other proteins. Consequently, there would be significantly more protein–protein interactions, elevated gene ontology homogeneity and co-expression levels. Analyses of microbial co-existing relationships in the human and environment microbiomes [111, 112, 117] or spatial neighborhood [118] will provide useful reference resources to establish statistical significance of any novel pairs of bacterial groups. Drug disease network [119] can also be re-evaluated to offer new options for novel development of therapeutics.

### Health monitoring by analyzing microbiome in the blood

Blood plasma has routinely been used to identify microbiome with the collection of bacteria and its products (e.g. nucleotide), for assessment of health status. The general assumption has been that nucleic acids originated mostly from gut microbiome with shedding into the blood. [120]. However, the existence of live microbiota in the blood circulation of apparently normal people was quite unexpected. For example, non-human small RNAs from *Proteobacteria* and *fungus Hypocreales* were detected in human blood samples [121]. An assay that detects and sequences plasma cell-free DNA (cfDNA) has been used to simultaneously monitor for infection and rejection in lung transplant recipients [122]. While the level of donor-derived cfDNA was strongly correlated with rejection, the level of cytomegalovirus-derived sequences cfDNA was indicative of infection. The small-molecular products from the gut microbiome can permeate the human serum and influence the rest of the human body [120]. Therefore, there are opportunities to investigate the role of blood microbiome in the disease process and the role of metabolome.

Some functional significance of microbiome in the blood circulation have recently been revealed [120]. For example, microvesicles which are laden with exogenous microbial RNA have the potential to function as signaling molecules in human plasma [123]. Non-human small RNAs from *Proteobacteria* and fungus *Hypocreales* were also detected in human blood samples [121]. Monitoring of nonhuman cell free DNA revealed undiagnosed infection which complicated prognosis [8]. Since alcohol has been found to increase intestinal permeability and it is a risk factor for endotoxemia, exploring the role of non-coding microbial RNAs in alcoholic liver disease is a natural direction for investigations, after adjusting for host genetic and other factors in alcohol elimination [124]. Cirrhosis dysbiosis ratio which is derived from stool microbiome can actually be tested to determine whether results obtained from blood microbiome are comparable to those from stool. For example, among 286 chronically HIV-infected individuals and AIDS patients, the LPS and 16S rDNA levels, and the percentage of CD8+, CD38+ and HLA-DR+ T cells were significantly higher than that from the uninfected controls [6]. Along with our previous reports [8, 9], health/disease status monitoring by assessing blood microbiome can be highly rewarding.

## Conclusions

In this review, we have provided an overview of gut microbiome and some relevant research questions. Certainly, the use of molecular genetics, high-throughput procedures, bioinformatics and modeling can complement conventional techniques and fill existing knowledge gaps. How to make good use of the knowledge to benefit patient care and to optimize treatment plans (e.g. personalized) are valuable topics for investigation. Interventions such as long-term diet modification on controlling health risks [30] are appealing and cost- effective approaches. As long as sequencing cost can be further reduced, regular monitoring of personalized genome, transcriptome and microbiome will become more applicable in the near future. Collaborations among universities, hospitals and biotechnology companies can significantly enhance achieving these goals.
