# Correction to: Involvement of gut microbiome in human health and disease: brief overview, knowledge gaps and research opportunities

**DOI:** 10.1186/s13099-019-0339-0

**Published:** 2019-11-21

**Authors:** Dachao Liang, Ross Ka‑Kit Leung, Wenda Guan, William W. Au

**Affiliations:** 1 Division of Genomics and Bioinformatics, CUHK-BGI Innovation Institute of Trans-omics Hong Kong, Hong Kong, SAR China; 2 State Key Laboratory of Respiratory Disease, National Clinical Research Center for Respiratory Disease, First Affiliated Hospital of Guangzhou Medical University, Guangzhou, Guangdong China; 3 University of Medicine and Pharmacy, Tirgu Mures, Romania; 4 Shantou University Medical College, Shantou, China; 5 Guangzhou Medical University, Guangzhou, Guangdong China

## Correction to: Gut Pathog (2018) 10:3 10.1186/s13099-018-0230-4

Following publication of the original article in Gut Pathogens [1], it was brought to our attention that Dr. Ross Ka Kit Leung’s affiliation was incomplete. Dr. Leung is appointed as an Adjunct Professor of Guangzhou Medical University from January 1st 2017 to December 31st 2019. He would like to correct here the affiliation and acknowledge his role at the Guangzhou Medical University when this article was published.

The correct information about the author affiliation is:

- Ross Ka-Kit Leung
- Guangzhou Medical University, Guangzhou, Guangdong, China
- State Key Laboratory of Respiratory Disease, National Clinical Research Center for Respiratory Disease, First Affiliated Hospital of Guangzhou Medical University, Guangzhou, Guangdong, China
